# Bis(triphenyl­stann­yl) thio­phene-2,5-dicarboxyl­ate

**DOI:** 10.1107/S1600536809020273

**Published:** 2009-06-06

**Authors:** Lichun Zhao, Jian Liang, Guihua Yue, Xin Deng, Ying He

**Affiliations:** a The Affiliated Ruikang Hospital of Guangxi Traditional Chinese Medical College, Nanning, Guangxi 530011, People’s Republic of China

## Abstract

Mol­ecules of the title compound, [Sn_2_(C_6_H_5_)_6_(C_6_H_2_O_4_S)], lie on inversion centres with the central thio­phene ring disordered equally over two orientations. The carboxyl­ate groups are approximately coplanar with the thio­phene ring [dihedral angle = 4.0 (1)°] and the Sn—O bond distance of 2.058 (4) Å is comparable to that in related organotin carboxyl­ates.

## Related literature

For background literature concerning organotin chemisty, see: Prabusankar & Murugavel (2004 ▶); Holmes (1989 ▶). For related structures, see: Pellei *et al.* (2008 ▶).
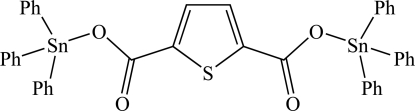


## Experimental

### 

#### Crystal data

[Sn_2_(C_6_H_5_)_6_(C_6_H_2_O_4_S)]
*M* *_r_* = 870.12
Monoclinic, 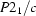

*a* = 10.1302 (10) Å
*b* = 18.699 (2) Å
*c* = 10.3584 (11) Å
β = 108.213 (2)°
*V* = 1863.8 (3) Å^3^
*Z* = 2
Mo *K*α radiation
μ = 1.44 mm^−1^
*T* = 298 K
0.21 × 0.11 × 0.06 mm

#### Data collection

Bruker SMART APEX CCD diffractometer
Absorption correction: multi-scan (*SADABS*; Sheldrick, 1996 ▶) *T*_min_ = 0.752, *T*_max_ = 0.919
9058 measured reflections
3281 independent reflections
2342 reflections with *I* > 2σ(*I*)
*R*_int_ = 0.040

#### Refinement

*R*[*F* ^2^ > 2σ(*F* ^2^)] = 0.052
*wR*(*F* ^2^) = 0.110
*S* = 1.02
3281 reflections
244 parameters
H-atom parameters constrained
Δρ_max_ = 0.77 e Å^−3^
Δρ_min_ = −0.63 e Å^−3^

### 

Data collection: *SMART* (Siemens, 1996 ▶); cell refinement: *SAINT* (Siemens, 1996 ▶); data reduction: *SAINT*; program(s) used to solve structure: *SHELXS97* (Sheldrick, 2008 ▶); program(s) used to refine structure: *SHELXL97* (Sheldrick, 2008 ▶); molecular graphics: *SHELXTL* (Sheldrick, 2008 ▶); software used to prepare material for publication: *SHELXTL*.

## Supplementary Material

Crystal structure: contains datablocks I, global. DOI: 10.1107/S1600536809020273/bi2371sup1.cif

Structure factors: contains datablocks I. DOI: 10.1107/S1600536809020273/bi2371Isup2.hkl

Additional supplementary materials:  crystallographic information; 3D view; checkCIF report

## Figures and Tables

**Fig. 1.**
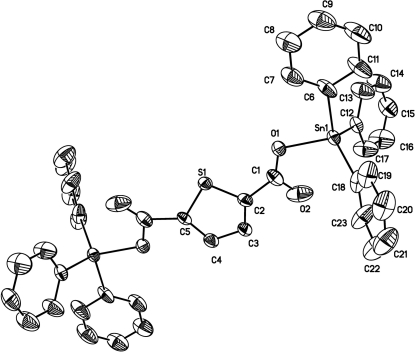
Molecular structure showing 30% probability displacement ellipsoids, with H atoms are omitted. Unlabelled atoms are related to labelled atoms by the symmetry code: 2-x, -y, 1-z. The symmetry-generated component of the disordered thiophene ring is not shown.

## supplementary crystallographic information

### Comment

The structural diversity of organotin carboxylates is well recognized and a wide variety of coordination geometries have been reported (Holmes, 1989). It is generally believed that a combination of steric and electronic factors determine the specific structure adapted by a particular organotin carboxylate (Prabusankar & Murugavel, 2004). This is supported through the observation of monomeric, dimeric, tetrameric, oligomeric ladder, cyclic, and drum structures. Furthermore, it has been reported that the size of the carboxylic acids used and the stoichiometry of the reactants play an important role in the formation of solid-state frameworks.

### Experimental

The reaction was carried out under a nitrogen atmosphere. Thiophene-2,5-dicarboxylic acid (10 mmol) and sodium ethoxide (20 mmol) were added to a stirred solution of benzene (50 ml) in a three-necked flask and stirred for 0.5 h. Triphenyltin chloride (20 mmol) was then added and the reaction mixture was stirred for 6 h at room temperature. The resulting clear solution was evaporated under vacuum. The product was crystallized from dichloromethane to yield colourless blocks of the title compound. Elemental analysis: calculated C 57.97, H 3.71 %; found: C 57.68, H 3.55 %.

### Refinement

H atoms were placed in geometrically idealized positions (C—H = 0.93 Å) and treated as riding on their parent atoms, with *U*_iso_(H) = 1.2 *U*_eq_(C).

### Crystal data

**Table:**

[Sn_2_(C_6_H_5_)_6_(C_6_H_2_O_4_S)]	*F*(000) = 864
*M**_r_* = 870.12	*D*_x_ = 1.550 Mg m^−^^3^
Monoclinic, *P*2_1_/*c*	Mo *K*α radiation, λ = 0.71073 Å
Hall symbol: -P 2ybc	Cell parameters from 3030 reflections
*a* = 10.1302 (10) Å	θ = 2.4–25.2°
*b* = 18.699 (2) Å	µ = 1.44 mm^−^^1^
*c* = 10.3584 (11) Å	*T* = 298 K
β = 108.213 (2)°	Needle, colorless
*V* = 1863.8 (3) Å^3^	0.21 × 0.11 × 0.06 mm
*Z* = 2	

### Data collection

**Table:**

Bruker SMART APEX CCD diffractometer	3281 independent reflections
Radiation source: fine-focus sealed tube	2342 reflections with *I* > 2σ(*I*)
graphite	*R*_int_ = 0.040
φ and ω scans	θ_max_ = 25.0°, θ_min_ = 2.1°
Absorption correction: multi-scan (*SADABS*; Sheldrick, 1996)	*h* = −12→6
*T*_min_ = 0.752, *T*_max_ = 0.919	*k* = −22→20
9058 measured reflections	*l* = −11→12

### Refinement

**Table:**

Refinement on *F*^2^	Primary atom site location: structure-invariant direct methods
Least-squares matrix: full	Secondary atom site location: difference Fourier map
*R*[*F*^2^ > 2σ(*F*^2^)] = 0.052	Hydrogen site location: inferred from neighbouring sites
*wR*(*F*^2^) = 0.110	H-atom parameters constrained
*S* = 1.02	*w* = 1/[σ^2^(*F*_o_^2^) + (0.0214*P*)^2^ + 10.153*P*] where *P* = (*F*_o_^2^ + 2*F*_c_^2^)/3
3281 reflections	(Δ/σ)_max_ = 0.001
244 parameters	Δρ_max_ = 0.77 e Å^−^^3^
0 restraints	Δρ_min_ = −0.63 e Å^−^^3^

### Special details

**Table:**

Geometry. All e.s.d.'s (except the e.s.d. in the dihedral angle between two l.s. planes) are estimated using the full covariance matrix. The cell e.s.d.'s are taken into account individually in the estimation of e.s.d.'s in distances, angles and torsion angles; correlations between e.s.d.'s in cell parameters are only used when they are defined by crystal symmetry. An approximate (isotropic) treatment of cell e.s.d.'s is used for estimating e.s.d.'s involving l.s. planes.
Refinement. Refinement of *F*^2^ against ALL reflections. The weighted *R*-factor *wR* and goodness of fit *S* are based on *F*^2^, conventional *R*-factors *R* are based on *F*, with *F* set to zero for negative *F*^2^. The threshold expression of *F*^2^ > σ(*F*^2^) is used only for calculating *R*-factors(gt) *etc*. and is not relevant to the choice of reflections for refinement. *R*-factors based on *F*^2^ are statistically about twice as large as those based on *F*, and *R*- factors based on ALL data will be even larger.

### Fractional atomic coordinates and isotropic or equivalent isotropic displacement parameters (Å^2^)

**Table:**

	*x*	*y*	*z*	*U*_iso_*/*U*_eq_	Occ. (<1)
Sn1	0.66000 (5)	0.07183 (3)	0.78299 (5)	0.04647 (17)	
S1	0.9299 (4)	0.0123 (2)	0.4533 (4)	0.0479 (9)	0.50
O1	0.7511 (5)	0.0595 (3)	0.6325 (5)	0.0547 (13)	
O2	0.9220 (7)	0.0178 (4)	0.7991 (8)	0.110 (3)	
C1	0.8710 (9)	0.0308 (4)	0.6808 (10)	0.061 (2)	
C2	0.9687 (17)	−0.0005 (9)	0.6205 (18)	0.053 (4)	0.50
C3	1.0869 (15)	−0.0332 (8)	0.6787 (16)	0.056 (4)	0.50
H3	1.1227	−0.0425	0.7713	0.067*	0.50
C4	1.1513 (16)	−0.0519 (8)	0.5836 (15)	0.057 (4)	0.50
H4	1.2341	−0.0775	0.6067	0.068*	0.50
C5	1.0826 (16)	−0.0293 (8)	0.4493 (19)	0.048 (4)	0.50
C6	0.4678 (8)	0.1119 (5)	0.6546 (9)	0.074 (3)	
C7	0.4344 (10)	0.1137 (5)	0.5168 (10)	0.089 (3)	
H7	0.4985	0.0981	0.4752	0.107*	
C8	0.3042 (12)	0.1388 (6)	0.4372 (12)	0.107 (4)	
H8	0.2812	0.1401	0.3430	0.129*	
C9	0.2123 (12)	0.1611 (6)	0.4998 (14)	0.113 (4)	
H9	0.1275	0.1798	0.4471	0.136*	
C10	0.2392 (11)	0.1573 (7)	0.6336 (14)	0.123 (5)	
H10	0.1723	0.1709	0.6731	0.147*	
C11	0.3691 (9)	0.1327 (6)	0.7143 (12)	0.107 (4)	
H11	0.3894	0.1304	0.8082	0.128*	
C12	0.7680 (8)	0.1508 (4)	0.9236 (7)	0.0532 (19)	
C13	0.7151 (11)	0.2181 (5)	0.9169 (11)	0.100 (3)	
H13	0.6362	0.2302	0.8457	0.120*	
C14	0.7770 (12)	0.2686 (6)	1.0144 (12)	0.109 (4)	
H14	0.7375	0.3138	1.0089	0.131*	
C15	0.8898 (11)	0.2541 (6)	1.1140 (11)	0.091 (3)	
H15	0.9309	0.2884	1.1793	0.109*	
C16	0.9439 (12)	0.1896 (7)	1.1196 (12)	0.121 (4)	
H16	1.0244	0.1788	1.1899	0.145*	
C17	0.8848 (11)	0.1373 (5)	1.0241 (10)	0.102 (4)	
H17	0.9267	0.0927	1.0301	0.123*	
C18	0.6293 (11)	−0.0309 (5)	0.8534 (9)	0.072 (3)	
C19	0.4946 (13)	−0.0581 (6)	0.8128 (10)	0.101 (3)	
H19	0.4219	−0.0301	0.7600	0.121*	
C20	0.4668 (15)	−0.1284 (7)	0.8514 (12)	0.115 (4)	
H20	0.3770	−0.1465	0.8293	0.138*	
C21	0.5790 (17)	−0.1672 (7)	0.9219 (13)	0.127 (5)	
H21	0.5634	−0.2144	0.9418	0.152*	
C22	0.7120 (16)	−0.1434 (7)	0.9663 (12)	0.130 (5)	
H22	0.7840	−0.1725	1.0172	0.156*	
C23	0.7362 (14)	−0.0732 (6)	0.9321 (10)	0.110 (4)	
H23	0.8258	−0.0547	0.9629	0.132*	

### Atomic displacement parameters (Å^2^)

**Table:**

	*U*^11^	*U*^22^	*U*^33^	*U*^12^	*U*^13^	*U*^23^
Sn1	0.0405 (3)	0.0537 (3)	0.0483 (3)	0.0019 (3)	0.0182 (2)	−0.0062 (3)
S1	0.040 (2)	0.052 (2)	0.054 (3)	0.0089 (19)	0.0192 (19)	0.0058 (19)
O1	0.050 (3)	0.062 (3)	0.060 (3)	0.008 (3)	0.028 (3)	0.005 (3)
O2	0.092 (5)	0.082 (5)	0.118 (6)	0.012 (4)	−0.023 (5)	0.006 (4)
C1	0.048 (5)	0.061 (5)	0.080 (6)	−0.002 (4)	0.026 (5)	−0.017 (5)
C2	0.042 (10)	0.055 (10)	0.062 (11)	0.012 (8)	0.018 (9)	0.000 (8)
C3	0.049 (9)	0.065 (10)	0.057 (10)	0.017 (8)	0.020 (8)	0.002 (8)
C4	0.046 (9)	0.064 (10)	0.059 (10)	0.015 (8)	0.015 (8)	−0.002 (8)
C5	0.040 (9)	0.047 (10)	0.064 (12)	0.010 (8)	0.025 (9)	−0.005 (9)
C6	0.053 (5)	0.082 (6)	0.079 (6)	0.014 (5)	0.010 (5)	−0.042 (5)
C7	0.069 (6)	0.092 (7)	0.090 (7)	0.017 (5)	0.003 (6)	−0.041 (6)
C8	0.087 (8)	0.110 (9)	0.099 (8)	0.020 (7)	−0.008 (7)	−0.038 (7)
C9	0.080 (8)	0.111 (9)	0.121 (10)	0.026 (7)	−0.010 (8)	−0.038 (8)
C10	0.074 (7)	0.142 (11)	0.134 (11)	0.040 (7)	0.006 (8)	−0.056 (9)
C11	0.060 (6)	0.134 (9)	0.111 (8)	0.032 (6)	0.007 (6)	−0.055 (7)
C12	0.050 (4)	0.061 (5)	0.055 (5)	−0.002 (4)	0.025 (4)	−0.011 (4)
C13	0.090 (7)	0.081 (7)	0.104 (8)	0.019 (6)	−0.004 (6)	−0.039 (6)
C14	0.098 (8)	0.086 (7)	0.119 (9)	0.012 (7)	−0.001 (8)	−0.043 (7)
C15	0.082 (7)	0.092 (8)	0.092 (8)	−0.016 (6)	0.019 (6)	−0.046 (6)
C16	0.099 (9)	0.110 (9)	0.111 (9)	0.002 (8)	−0.031 (7)	−0.031 (8)
C17	0.089 (7)	0.081 (7)	0.096 (8)	0.015 (6)	−0.030 (6)	−0.024 (6)
C18	0.090 (7)	0.079 (6)	0.053 (5)	−0.033 (6)	0.033 (5)	−0.012 (5)
C19	0.121 (9)	0.104 (8)	0.080 (7)	−0.043 (7)	0.035 (7)	−0.015 (6)
C20	0.130 (11)	0.116 (10)	0.096 (9)	−0.064 (9)	0.033 (8)	−0.014 (7)
C21	0.149 (13)	0.119 (11)	0.102 (10)	−0.045 (10)	0.023 (10)	0.019 (8)
C22	0.151 (13)	0.113 (10)	0.103 (9)	−0.036 (9)	0.006 (9)	0.020 (8)
C23	0.146 (11)	0.092 (8)	0.072 (7)	−0.044 (8)	0.006 (7)	0.019 (6)

### Geometric parameters (Å, °)

**Table:**

Sn1—O1	2.058 (4)	C10—H10	0.930
Sn1—C18	2.112 (9)	C11—H11	0.930
Sn1—C6	2.121 (9)	C12—C17	1.333 (11)
Sn1—C12	2.122 (7)	C12—C13	1.359 (11)
S1—C2	1.669 (18)	C13—C14	1.382 (12)
S1—C5	1.744 (15)	C13—H13	0.930
O1—C1	1.279 (9)	C14—C15	1.307 (13)
O2—C1	1.197 (10)	C14—H14	0.930
C1—C2	1.449 (17)	C15—C16	1.320 (14)
C2—C3	1.31 (2)	C15—H15	0.930
C3—C4	1.385 (19)	C16—C17	1.386 (13)
C3—H3	0.930	C16—H16	0.930
C4—C5	1.41 (2)	C17—H17	0.930
C4—H4	0.930	C18—C23	1.382 (14)
C5—C1^i^	1.560 (19)	C18—C19	1.392 (13)
C6—C7	1.361 (12)	C19—C20	1.428 (14)
C6—C11	1.386 (12)	C19—H19	0.930
C7—C8	1.400 (13)	C20—C21	1.354 (16)
C7—H7	0.930	C20—H20	0.930
C8—C9	1.356 (15)	C21—C22	1.355 (16)
C8—H8	0.930	C21—H21	0.930
C9—C10	1.328 (15)	C22—C23	1.402 (14)
C9—H9	0.930	C22—H22	0.930
C10—C11	1.399 (13)	C23—H23	0.930
			
O1—Sn1—C18	108.0 (3)	C6—C11—H11	120.0
O1—Sn1—C6	96.1 (3)	C10—C11—H11	120.0
C18—Sn1—C6	109.4 (4)	C17—C12—C13	117.1 (8)
O1—Sn1—C12	109.9 (2)	C17—C12—Sn1	123.0 (6)
C18—Sn1—C12	119.7 (3)	C13—C12—Sn1	119.9 (6)
C6—Sn1—C12	111.1 (3)	C12—C13—C14	120.9 (9)
C2—S1—C5	92.1 (7)	C12—C13—H13	119.6
C1—O1—Sn1	110.3 (5)	C14—C13—H13	119.6
O2—C1—O1	122.7 (8)	C15—C14—C13	121.4 (10)
O2—C1—C2	103.0 (11)	C15—C14—H14	119.3
O1—C1—C2	134.0 (11)	C13—C14—H14	119.3
C3—C2—C1	129.7 (16)	C14—C15—C16	118.2 (10)
C3—C2—S1	115.5 (12)	C14—C15—H15	120.9
C1—C2—S1	114.7 (12)	C16—C15—H15	120.9
C2—C3—C4	110.8 (15)	C15—C16—C17	122.2 (10)
C2—C3—H3	124.6	C15—C16—H16	118.9
C4—C3—H3	124.6	C17—C16—H16	118.9
C3—C4—C5	115.4 (15)	C12—C17—C16	120.2 (10)
C3—C4—H4	122.3	C12—C17—H17	119.9
C5—C4—H4	122.3	C16—C17—H17	119.9
C4—C5—S1	106.1 (13)	C23—C18—C19	118.9 (9)
C1^i^—C5—S1	122.5 (12)	C23—C18—Sn1	123.4 (7)
C7—C6—C11	118.9 (9)	C19—C18—Sn1	117.6 (8)
C7—C6—Sn1	123.0 (6)	C18—C19—C20	120.8 (12)
C11—C6—Sn1	117.9 (7)	C18—C19—H19	119.6
C6—C7—C8	120.4 (10)	C20—C19—H19	119.6
C6—C7—H7	119.8	C21—C20—C19	116.0 (12)
C8—C7—H7	119.8	C21—C20—H20	122.0
C9—C8—C7	118.9 (11)	C19—C20—H20	122.0
C9—C8—H8	120.6	C20—C21—C22	125.8 (13)
C7—C8—H8	120.6	C20—C21—H21	117.1
C10—C9—C8	122.3 (11)	C22—C21—H21	117.1
C10—C9—H9	118.8	C21—C22—C23	117.1 (13)
C8—C9—H9	118.8	C21—C22—H22	121.5
C9—C10—C11	119.3 (12)	C23—C22—H22	121.5
C9—C10—H10	120.4	C18—C23—C22	121.2 (12)
C11—C10—H10	120.4	C18—C23—H23	119.4
C6—C11—C10	120.1 (11)	C22—C23—H23	119.4

Symmetry codes: (i) −*x*+2, −*y*, −*z*+1.
